# Targeted Intermittent Treatment in Chronic Schizophrenia

**DOI:** 10.3389/fpsyt.2013.00013

**Published:** 2013-03-14

**Authors:** Adonis Sfera

**Affiliations:** ^1^South Coast Clinical TrialsAnaheim, CA, USA

## Introduction

Talking about non-continuous antipsychotic treatment in psychiatric practice in our time is tantamount to heresy. We have been perseverating in comparing psychiatric illness to diabetes and hypertension so much that we are having a hard time fathoming a different analogy; perhaps, lupus, asthma, rheumatoid arthritis, or cancer in which intermittent dosing for shorter period of time is acceptable.

In 1960s and 1970s, there was a lot of talk about targeting intermittent treatment throughout the duration of illness, rather than chronic and final phase of schizophrenia. Widespread use of antipsychotics is historically novel that only a small percentage phase four patients are being seen currently by average psychiatrists, but this number may grow in time. Reviewing the psychiatric literature from 1970s, it appears that continuous antipsychotic exposure were not always necessary at that time (Prien et al., 1973).

Even in our times there are advocates of non-continuous treatment after the first episode of schizophrenia, as about 50% of patients have remained stable after discontinuing medications in the second year of treatment (Harrow and Jobe, 2007). Also, the duration of antipsychotic treatment after a first episode of psychosis remains unclear. A comparison of evidence-based treatment guidelines from different countries developed on highest-quality criteria, yielded inconsistent recommendations regarding the duration of maintenance treatment (Gaebel et al., 2011).

## Tolerance to Antipsychotic Drugs?

Reviewing the current body of evidence, it appears that though the efficacy of antipsychotics in general is proven, a significant proportion of patients suffer from partial remission, symptom recurrence, or relapse, despite continuous antipsychotic treatment. This also applies to depot neuroleptic treatment, which minimizes adherence problems.

Several studies suggest that with careful clinical observation, substantial reduction of maintenance doses can, for many patients, lead to improvement in some areas of subjective and objective well-being and to a diminution of adverse effects (Kane et al., 1986).

Schizophrenic patients remain on antipsychotic (i.e., antidopaminergic) treatment for years, yet remarkably little is known about what happens to the dopamine function during ongoing treatment (Remington and Kapur, 2010). What is known is that in late stages of schizophrenia, antipsychotics become inefficient frequently, despite chronic continuous treatment.

There is evidence from preclinical as well as clinical studies that point to the build up of dopamine supersensitivity (Samaha et al., 2007) and tolerance to antipsychotics, leading to treatment failure over time.

Neuroleptic-induced supersensitivity psychosis (Chouinard et al., 1978; Chouinard and Jones, 1980) has been observed after withdrawal from antipsychotic drugs such as quetiapine (Margolese et al., 2002), clozapine (Ekblom et al., 1984; Tollefson et al., 1999), olanzapine (Llorca et al., 2001), haloperidol (Kahne, 1989), and fluphenazine enanthate (Chouinard and Jones, 1980).

Clinical data are available to suggest antipsychotic tolerance with continuous treatment (Stip et al., 1995). The late or chronic stages of schizophrenia are associated with higher antipsychotic doses and diminished clinical response, also suggesting tolerance (Remington et al., 1997; Yamin and Vaddadi, 2010).

**Figure 1:**
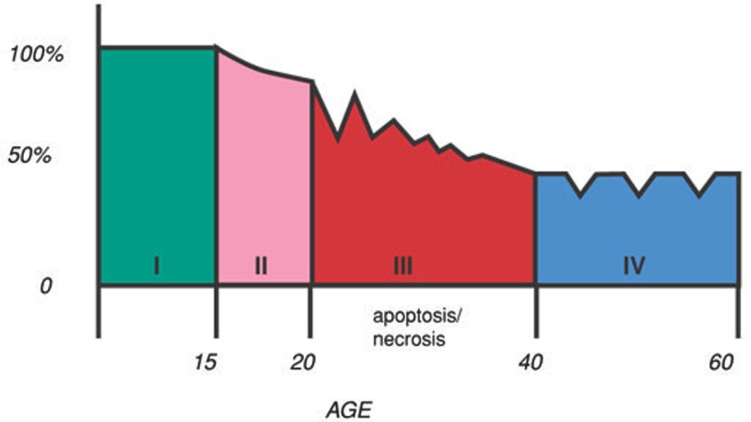
**Stages of schizophrenia**. Stage 1. The patient is fully functional in early part of life, and is virtually asymptomatic. Stage 2. Prodromal phase starts in teens. There may be odd behaviors and subtle negative symptoms. Stage 3. The acute phase of the illness occurs in twenties or thirties, with positive and negative symptoms. Stage 4. “Burn out” phase occurs in forties or fifties with prominent negative and cognitive symptoms.

Is it possible that in chronic “burn out” phase of schizophrenia (Figure 1) in which there is neuronal and synaptic loss, a targeted or intermittent antipsychotic treatment is more beneficial to the patient? In dementia, where there is extensive cerebral tissue loss, antipsychotics are being used only intermittently and for a shorter duration of time.

Just wondering if schizophrenic patients in late phases would benefit from a similar strategy. At this time, therapy using “as needed” antipsychotic medications is being discouraged in favor of continuous treatment, yet quite the opposite might be needed in this stage.

## Brain Changes in Long-Term Use of Antipsychotics

Recent data including animal studies, suggests that long-term antipsychotic treatment leads to global brain volume reduction (Ho et al., 2011).

In medicine, we are aware of many instances where target symptom improves by worsening other symptoms. Hormone therapy relieves menopausal symptoms but increases stroke risk. Non-steroidal anti-inflammatory drugs relieve pain, but increase the likelihood of duodenal ulcers and gastrointestinal tract bleeding. It is possible that although antipsychotics relieve psychosis and its attendant suffering, these drugs may not arrest the pathophysiologic processes underlying schizophrenia, and may even aggravate progressive brain tissue volume reductions (Ho et al., 2011).

## Conclusion

The last phase of schizophrenia or the “burn out” phase, like dementia, is characterized by extensive neuronal and synaptic loss. Since both chronic schizophrenia and prolonged antipsychotic treatment result in cerebral tissue loss, it is appropriate to revisit intermittent, targeted, or “as needed” use of antipsychotics at this stage.
